# Familial occurrences of cardiac wild-type transthyretin amyloidosis: a case series

**DOI:** 10.1093/ehjcr/ytae199

**Published:** 2024-04-18

**Authors:** Oscar M Westin, Tor S Clemmensen, Anne Tybjærg Hansen, Finn Gustafsson, Steen Hvitfeldt Poulsen

**Affiliations:** Department of Cardiology, Rigshospitalet, Copenhagen University Hospital, Blegdamsvej 9, 2100 Copenhagen, Denmark; Department of Cardiology, Aarhus University Hospital, Palle Juul-Jensens Boulevard 99, 8200 Aarhus, Denmark; Department of Clinical Biochemistry, Rigshospitalet, Copenhagen University Hospital, Blegdamsvej 9, 2100 Copenhagen, Denmark; Department of Cardiology, Rigshospitalet, Copenhagen University Hospital, Blegdamsvej 9, 2100 Copenhagen, Denmark; Department of Cardiology, Aarhus University Hospital, Palle Juul-Jensens Boulevard 99, 8200 Aarhus, Denmark

**Keywords:** Case report, Cardiac amyloidosis, Wild-type transthyretin, Screening, Heart failure

## Abstract

**Background:**

Cardiomyopathy caused by aggregation and deposition of transthyretin amyloid fibrils in the heart (ATTR-CM) is divided into a hereditary (ATTRv) and a wild-type (ATTRwt) forms. While ATTR-CM has been considered a rare disease, recent studies suggest that it is severely underdiagnosed and an important cause of heart failure in elderly patients. Familial occurrence is implicit in ATTRv, but it is not expected in ATTRwt.

**Case summary:**

We report a case series of two unrelated families each with two brothers diagnosed with ATTRwt. Genetic testing did not reveal mutations in the transthyretin gene. Family screening with electrocardiogram, echocardiography, and genetic testing did not raise any suspicion of ATTR in first-line family members.

**Discussion:**

Familial occurrence of a rare, non-hereditary disease is statistically unlikely. Two siblings in two different families diagnosed with ATTRwt highlight that the aetiology of ATTRwt is poorly understood, and that genetic factors distinct from mutations in the transthyretin gene, as well as environmental factors, might contribute to the pathogenesis. Identifying such factors might reveal new therapeutic targets. To investigate this further, clinicians need to be aware of the possibility of familial occurrence of ATTRwt.

**Learning points**

- The existence of unknown genetic or environmental factors precipitating wild-type transthyretin amyloidosis is plausible.
- Diagnostic work-up for cardiac amyloidosis should be considered in siblings of patients with wild-type transthyretin amyloidosis, if they present with cardiac symptoms.

## Introduction

Wild-type transthyretin amyloidosis (ATTRwt) is increasingly recognized as an important cause of heart failure, arrhythmia, and conduction disorders in elderly patients.^1^ Breakthroughs in disease-modifying treatment have sparked widespread interest in the condition, and straightforward diagnostic algorithms using non-invasive imaging have facilitated the diagnostic work-up, leading to increased diagnostic rates.^2^ Cardiac societies endorse common guidelines for the diagnosis and management of cardiac amyloidosis (CA), unifying diagnostic and therapeutic approaches internationally.^3^ Nonetheless, the cause of ATTRwt remains unknown. In the absence of amyloidogenic variants of the transthyretin (TTR) gene, the ATTRwt pathophysiology should derive from interactions between translated TTR and its environment. The amount of such possible interactions is extensive, including post-translational modifications,^4^ interactions with circulating proteins (e.g. extracellular chaperones and proteolytic enzymes^5^), interactions with the extracellular matrix and the basement membrane,^6,7^ auxiliary proteins included in amyloid fibrils (e.g. serum amyloid protein^8^), as well as local mechanical forces (e.g. shear stress^9^). Considering these possibilities, the existence of genetic contributors to ATTRwt seems plausible. If so, should screening for CA be considered in siblings of patients with ATTRwt?

## Summary figure

**Figure:**
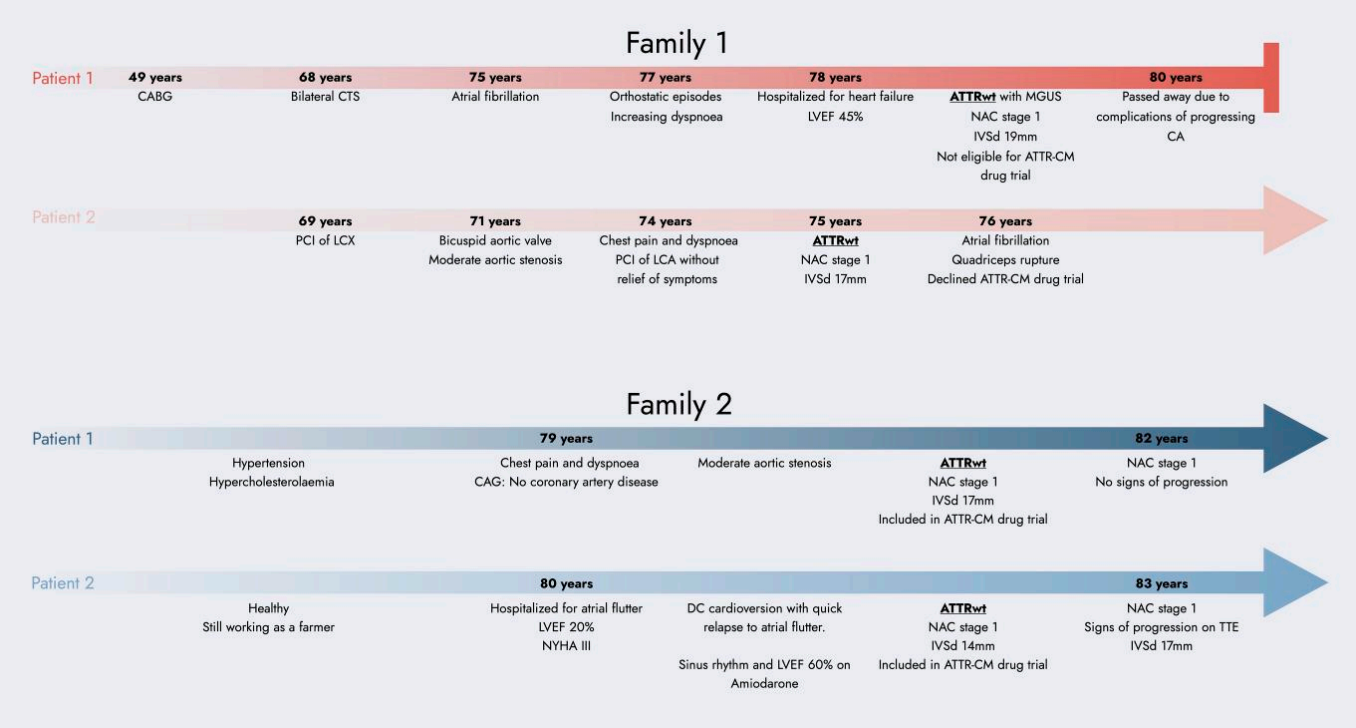


## Case summaries

### Family 1: Patient 1

**Figure 1:**
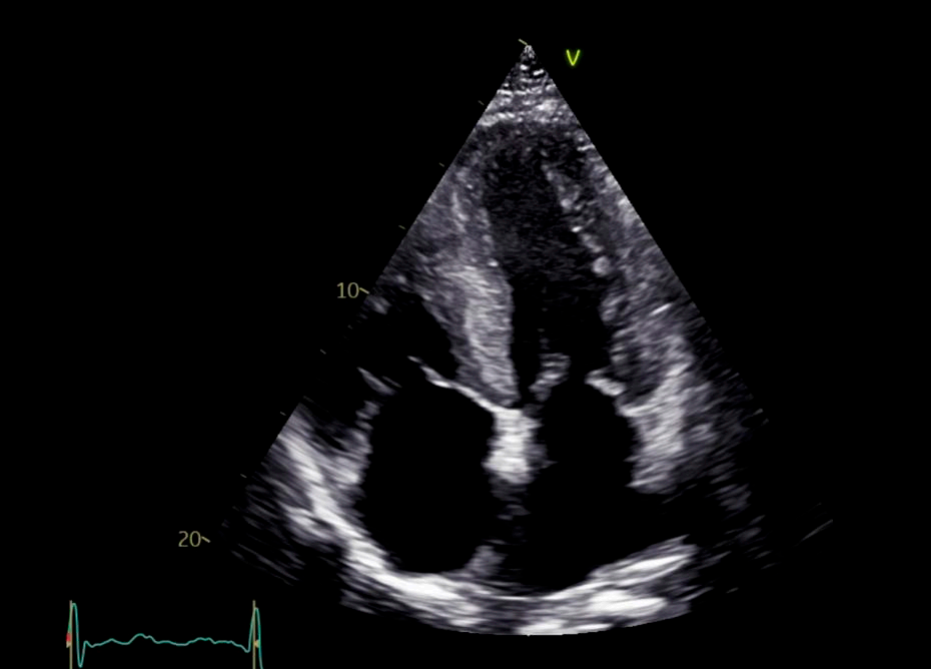
Family 1, Patient 1: transthoracic echocardiography in a four-chamber view, showing severe wall thickening and atrial dilatation.

**Figure 2:**
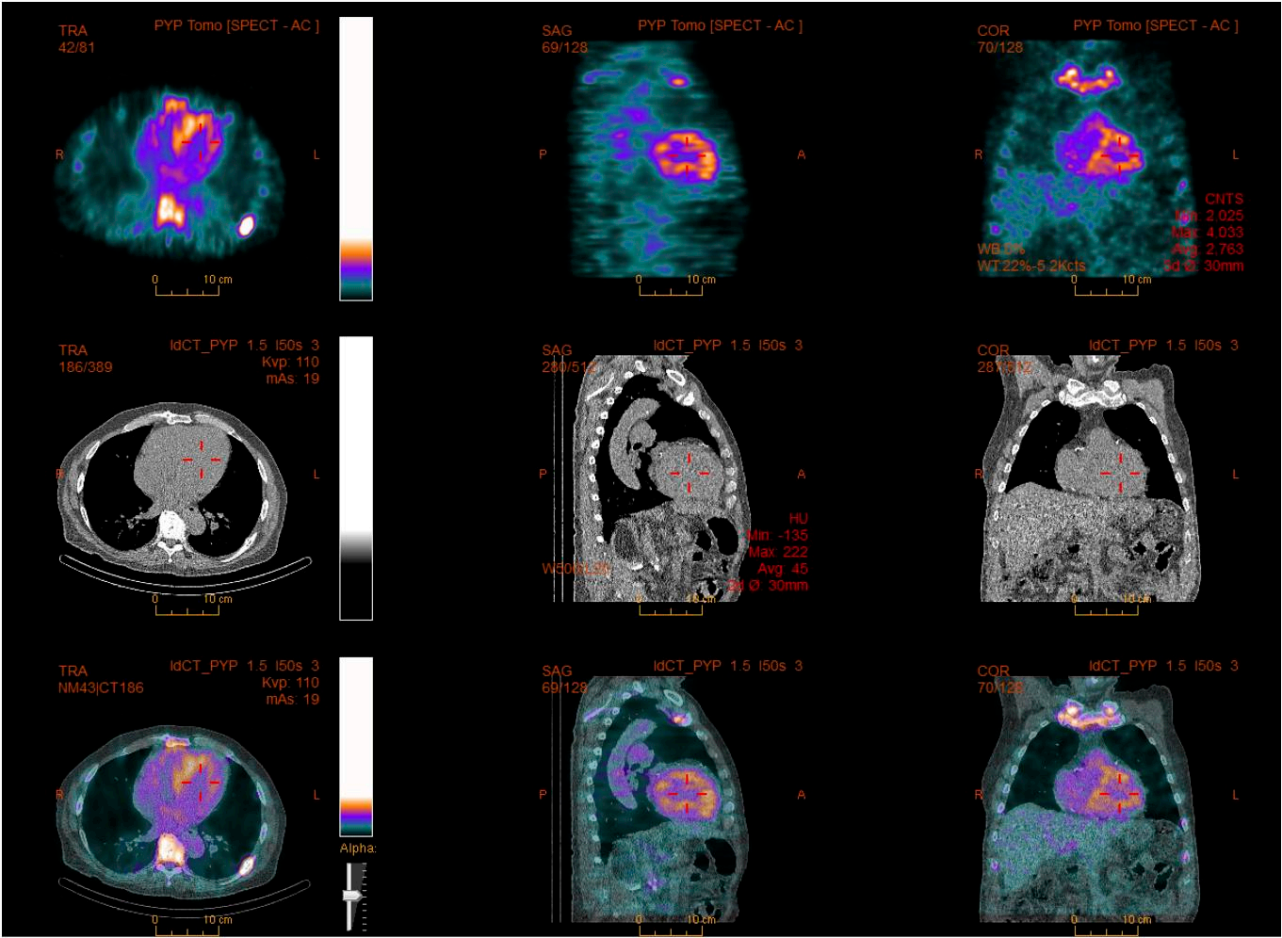
Family 1, Patient 1: SPECT/CT showing a myocardial uptake of the radiotracer.

A 78-year-old Caucasian man with a history of hypertension, Type 2 diabetes mellitus, dyslipidaemia, atrial fibrillation, peripheral artery disease, and previous coronary artery bypass graft surgery was hospitalized due to increasing dyspnoea and peripheral oedema. His medical history included previous surgery for bilateral carpal tunnel syndrome 10 years earlier, a typical red flag for amyloidosis.^1^ The patient reported multiple falls possibly due to orthostatic hypotension during the past year. Diagnostic work-up showed a normal heart rate, blood pressure, and saturation, the stethoscopy of the lungs was without crepitations, and the chest radiogram was without signs of pulmonary congestion. Intravenous loop diuretic therapy was initiated, followed by transthoracic echocardiography (TTE) demonstrating severe, concentric left ventricular hypertrophy. The interventricular septum thickness in diastole (IVSd) was 19 mm. The left ventricular ejection fraction (LVEF) was 45%. A global longitudinal strain (GLS) analysis showed reduced strain with an apical sparing pattern. The diastolic function was impaired, *E*/*e*′ was 25, and both atria were enlarged. The right ventricle was thickened, and the systolic function was impaired with a tricuspid annular plane systolic excursion (TAPSE) of 10 mm. There were no valvular pathologies or any pericardial effusion. After discharge, a technetium-99m pyrophosphate scintigraphy with single-photon emission computer tomography (SPECT) was performed, which showed Perugini Grade 2 uptake in the myocardium. Sequencing of the genes *APOA1*, *APOA2*, *APOA4*, *CST3*, *FGA*, *GSN*, *LYZ*, and *TTR* was all found negative for genetic variants associated with amyloidosis. DNA was extracted from peripheral blood using standard methods. Next-generation sequencing (NGS) techniques were used for the genetic screening. Exome/RefSeq with the spike-in panel from TWIST Bioscience (South San Francisco, CA, USA) was used. DNA was prepared according to the TWIST NGS workflow protocol and sequenced on a Novaseq System (Illumina, CA, USA). Poor-quality reads were filtered. Alignment to hg19 was performed with Burrows–Wheeler Alignment v. 0.7.6a, and GATK V.4.1.0.0 was used for variant calling, after performing all steps as described in best practice using workflow descriptive language on Cromwell v.36. A minimum average coverage of 30× was guaranteed (complex genomic regions were Sanger-sequenced). The serum-free light chain ratio (kappa/lambda ratio) was normal, but monoclonal immunoglobulin M was found in serum. In collaboration with the Department of Hematology, the patient was diagnosed with ATTRwt with a concomitant monoclonal gammopathy of undetermined significance. At diagnosis, the patient was in National Amyloidosis Center (NAC^10^) Stage I [N-terminal pro B-type natriuretic peptide (NT-proBNP) 2046 pg/mL (normal range: <450 pg/mL in adults ≥75 years), estimated glomerular filtration rate (eGFR) 52 mL/1.73 m^2^ (normal range: >90 mL/1.73 m^2^)]. The patient was screened for participation in a randomized controlled trial on disease-modifying treatment for ATTR-CM but was ineligible due to mild thrombocytopenia. The patient had recurring falls and suffered a stable cervical fracture before passing away a year later, ultimately due to complications of progressing CA (*Figures 1* and *2*).

### Family 1: Patient 2

A 75-year-old Caucasian man was referred for further cardiac evaluation at a university hospital due to persistent chest pain and dyspnoea. The patient had a bicuspid aortic valve with moderate stenosis and ischaemic heart disease (IHD) with percutaneous intervention on the circumflex coronary artery 6 years earlier and on the left coronary artery, 6 months earlier without symptomatic improvement. The lung stethoscopy results were normal, but there was a systolic murmur on heart stethoscopy. Transthoracic echocardiography showed a severe concentric wall thickening of the left ventricle with an IVSd of 17 mm, LVEF 60% but poor longitudinal systolic function with a GLS of −6.8% and an apical sparing pattern, diastolic dysfunction with *E*/*e*′ 34, and bi-atrial dilatation. The bicuspid aortic valve was moderately stenotic (a peak gradient of 44 mmHg and a calculated aortic valve area of 1.1 cm^2^). The right ventricle was thickened but with a normal TAPSE of 26 mm. There was no pericardial effusion. Blood samples showed normal cardiac troponins, increased NT-proBNP 2465 ng/L, and eGFR 54 mL/1.73 m^2^ corresponding to NAC Stage I. The serum kappa/lambda ratio was normal, and there was no evidence of monoclonal proteins in the serum or urine. A ^99m^Tc-3,3-diphosphono-1,2-propanodicarboxylic acid (DPD) scintigraphy with SPECT showed Perugini Grade 3 uptake in the myocardium. Genetic screening, using NGS (Illumina), NimbleGen DNA capture with a minimum average coverage of 30×, did not show disease-causing genetic variants in *TTR* (NM_000371.3), and the patient was diagnosed with ATTRwt. The patient was invited to participate in a randomized controlled trial on disease-modifying treatment for ATTR-CM but declined participation. Seven weeks after the ATTRwt diagnosis, the patient suffered a right-sided quadriceps rupture while walking on stairs. One year later, the patient developed atrial fibrillation but remained in NAC Stage I.

### Family screening in the first family

**Figure 3:**
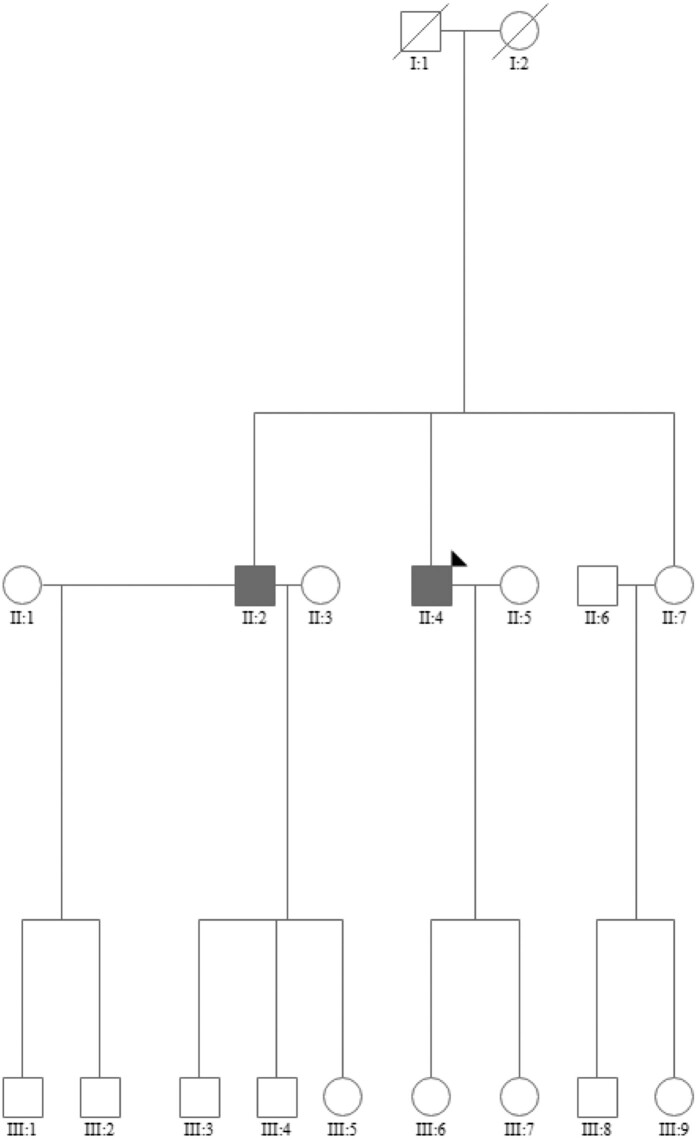
Genetic evaluation of Family 1.

The two siblings had one sister (*Figure 3*, II:7). Furthermore, the second patient had two children; a daughter aged 48 years (III:6) and a daughter aged 53 years (III:7). These three relatives were screened for ATTR by electrocardiogram (ECG), echocardiography, and genetic testing of *TTR* without detecting red flags or genetic variants raising suspicion for ATTR.

### Family 2: Patient 1

A 79-year-old Caucasian man was referred for coronary angiography due to dyspnoea and angina pectoris. He had a history of hypertension and hypercholesterolaemia but was otherwise in good health and had been a farmer for all his work life. His physical examination was unremarkable, with normal stethoscopy of the heart and lungs and no peripheral oedema. No significant coronary artery disease was detected; however, TTE showed severe concentric LV wall thickening with an IVSd of 17 mm and a thickening of the right ventricle. The LVEF was 55% and GLS −10.0% with an apical sparing pattern. The diastolic function was impaired, and *E*/*e*′ was 26. The aortic valve was moderately stenotic (a peak gradient of 40 mmHg and a calculated aortic valve area of 1.1 cm^2^). There was no pericardial effusion. Blood samples showed increased NT-proBNP of 1390 pg/mL and cardiac troponin T of 60 ng/L (normal range: <14 ng/L) and preserved eGFR of 75 mL/1.73 m^2^. The serum kappa/lambda ratio was normal, and there was no evidence of monoclonal proteins in the serum. A ^99m^Tc-DPD scintigraphy with SPECT showed Perugini Grade 3 uptake in the myocardium. Genetic screening, using NGS (Illumina), NimbleGen DNA capture with a minimum average coverage of 30×, did not show disease-causing genetic variants in *TTR* (NM_000371.3), and the patient was diagnosed with ATTRwt. The patient had an Achilles tendon rupture 12 months after the diagnosis. The patient has been participating in a randomized controlled trial on disease-modifying treatment for ATTR-CM for 3 years, and the ATTR diagnosis has been confirmed by endomyocardial biopsy. In this period, he has remained in NAC Stage I without significant ATTR progression on echocardiography.

### Family 2: Patient 2

**Figure 4:**
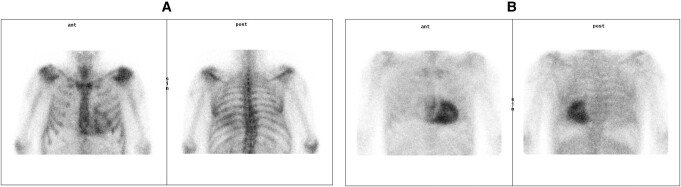
(*A*) Family 2, Patient 2: scintigraphy showing Perugini Grade 2 uptake. (*B*) Family 2, Patient 1: Scintigraphy showing Perugini Grade 3 uptake.

An 80-year-old Caucasian man was referred due to palpitations, dizziness, and dyspnoea. He was previously healthy without medication and still working as a farmer. At the time of admission, he was in NYHA Class III. Slight basal crepitations were heard on lung stethoscopy, but there was no peripheral oedema. Heart auscultation was normal, as also tachycardia. The ECG showed atrial flutter with a rate of 139 beats/min. Transthoracic echocardiography showed an LVEF of 20%, a moderate concentric LV wall thickening of 13–14 mm and an enlarged left atrium of 100 mL, no valvular pathology, and no pericardial effusion. A transoesophageal echocardiography ruled out thrombi, and the patient was cardioverted into sinus rhythm with immediate symptomatic improvement. Subsequently, the LVEF increased to 35%, but Troponin T and NT-ProBNP remained elevated at 51 ng/L and 1783 pg/mL, respectively. A coronary angiogram showed normal vessels. The serum kappa/lambda ratio was normal, and there was no evidence of monoclonal proteins in the serum or urine. A ^99m^Tc-DPD scintigraphy with SPECT showed Perugini Grade 2 uptake in the myocardium. Genetic screening, using NGS (Illumina), NimbleGen DNA capture with a minimum average coverage of 30×, did not show variants with known significance in *TTR* (NM_000371.3), and the patient was diagnosed with ATTRwt. The atrial flutter and fibrillation reoccurred, and amiodarone treatment was initiated. After stabilizing the heart rhythm, the LVEF improved to 60%, the diastolic function improved with an *E*/*e*′ of 7, and NT-proBNP decreased to 562 pg/mL. The patient has been participating in a randomized controlled trial on disease-modifying treatment for ATTR-CM for 3.5 years, and the ATTR diagnosis has been confirmed by endomyocardial biopsy. During follow-up, the patient has remained in NAC Stage I and has had surgery for carpal tunnel syndrome. The LV hypertrophy has showed progression to a wall thickness of 17 mm (*Figure 4*).

### Family and genetic screening in the second family

After the ATTRwt diagnosis was established in both siblings, additional genetic testing was performed in both patients using NGS (Illumina) with a minimum coverage of 10×. The panels and genes tested were:

PanelApp: *FGA*, *LYZ*, *NLRP3*, *APOC2*, *GSN*, *APOC3*, *APOA2*, *CST3*, *APOA1*, *TTR*, *B2M*.

VarSeq panel: *B2M*, *SAA1*, *OSMR*, *GPNMB*, *IL31RA*, *ITM2B*, *APP*, *CST3*, *TTR*, *FGA*, *APOA1*, *LYZ*, *GSN*.

HP:0011034: *APOA1*, *APOE*, *APP*, *ASXL1*, *B2M*, *CCND1*, *COL7A1*, *CST3*, *FGA*, *GSN*, *IL31RA*, *ITM2B*, *KIT*, *LIG4*, *LYZ*, *MEFV*, *MMP1*, *NLRP1*, *NLRP3*, *OSMR*, *POLA1*, *PRNP*, *PSEN2*, *RET*, *SAA1*, *SLC7A7*, *SRSF2*, *TET2*, *TNFRSF1A*, *TTR*.

**Figure 5:**
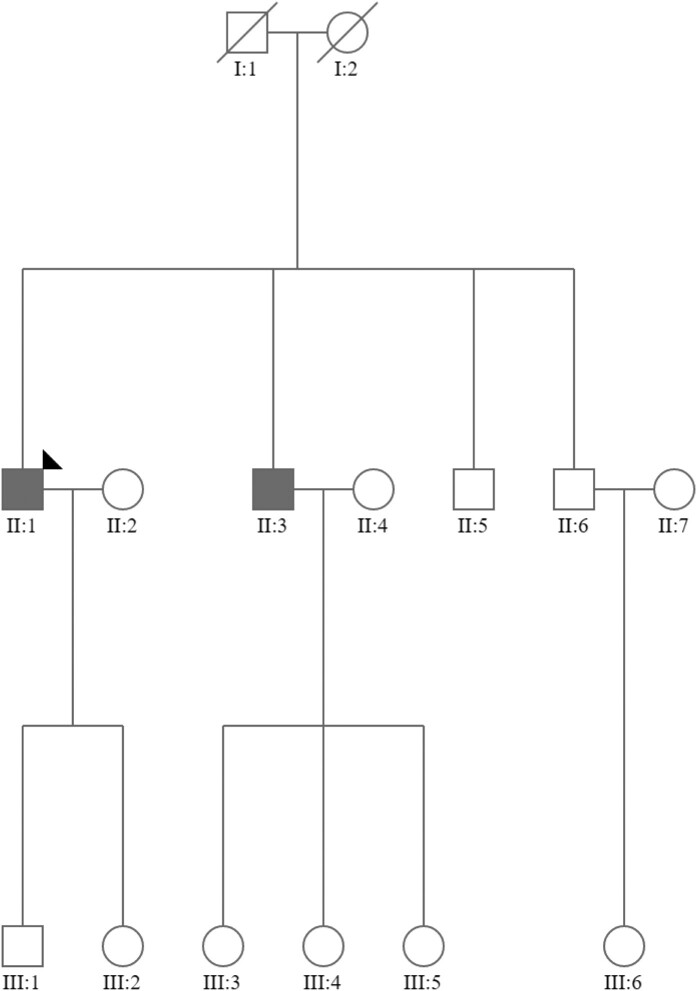
Genetic evaluation of Family 2.

We did not identify any genetic variants with known significance in any of the tested genes. Subsequently, a full family assessment with echocardiographic and ECG screening, in addition to genetic testing, was conducted. None of the children to the index patients had red flags or genetic variants associated with ATTR (*Figure 5*).

## Discussion

Familial occurrence of a rare, non-hereditary disease is statistically improbable. The rareness of ATTRwt has been re-evaluated during past years, as screening studies indicate that the condition is considerably more prevalent among elderly patients with heart disease than previously anticipated.^11–13^ Still, the finding of two cases of ATTRwt in the same family is unexpected. In this case series, we report two instances where two brothers were diagnosed with ATTRwt, highlighting that ATTRwt aetiology is poorly understood and that biological factors apart from transthyretin, as well as environmental factors, might contribute to the pathogenesis. While ‘new’ variants of varying clinical relevance are continuously uncovered, extensive genetic evaluation of the patients revealed no genetic variation in *TTR*. The described patients exhibited a cardiac phenotype, as is expected in ATTRwt. All patients met echocardiographic criteria for non-invasive/invasive diagnosis of CA, and this was why cardiac magnetic resonance imaging was not performed. In the absence of neurological symptoms, the patients were not investigated for signs of polyneuropathy. Disease-modifying drugs for ATTRwt are not reimbursed in our country, and due to their high cost, inclusion in clinical trials is currently the only way for patients to access these drugs. In accordance with international guidelines, the management of cardiac symptoms in these patients mainly relied on low-dose loop diuretics to maintain euvolaemia, while avoiding an exacerbation of orthostatism, as well as avoiding incorrect or unsuitable treatment.^3^

Genes determine the amino acid sequence in proteins. However, during the journey from the unfolded polypeptide chain to the functional native structure, proteins coexist in unfolded, partially unfolded, and folded states, governed by complex thermodynamic equilibria and kinetic barriers. Chaperones protect the unfolded protein from unwanted interactions with the environment and use energy to bind and release the polypeptide, guiding it through the folding process.^14,15^ In ATTR amyloidosis, protein homeostasis alteration has been suggested to be as important as point mutations in *TTR*, with patients exhibiting higher proteolytic activity and an over-representation of extracellular chaperones, interpreted as possible coping mechanisms.^5^

Even if no genetic variation is present and transthyretin has been successfully synthesized, post-translational modifications such as age-related oxidative modifications have been suggested to alter aggregation propensity.^4^ Furthermore, changes in the environment (e.g. the rate of protein synthesis, chaperone levels, pH, temperature, metabolites, and metal ions) or contact with protein aggregates or certain surfaces can trigger protein misfolding.^15,16^

Apart from the precursor protein itself, all amyloid fibrils contain the glycoprotein serum amyloid P-component, and various extracellular matrix components (e.g. Collagen IV, laminin, and apolipoproteins) are also commonly found in the amyloid deposits. The extracellular matrix characteristics are influenced by ageing, possibly affecting fibrillogenesis initiation.^17–19^ Finally, locally acting forces (e.g. shear stress and mechanic agitation) seem to play a part in where amyloid is deposited.^9^

Consequently, the existence of genetically determined amyloidogenic interactions between transthyretin and its environment, not confined to *TTR*, is plausible as evident by the presented cases. Understanding such interactions is necessary to elucidate the causes of ATTRwt and could reveal potential therapeutic targets. In the quest for identifying these interactions, studying siblings with ATTRwt might be pivotal, and this is why clinicians must be aware of this possibility.

Current international guidelines recommend diagnostic work-up for CA in patients with increased cardiac wall thickness and red flags for CA.^1^ Pre-symptomatic screening should be offered to first-degree relatives of patients with variant transthyretin amyloidosis, and active monitoring is important to detect early markers of disease penetrance in carriers of TTR variants. Analogous to this, diagnostic work-up for ATTRwt could be warranted in siblings of patients with ATTRwt if they present cardiac symptoms or conditions associated with ATTR, e.g. carpal tunnel syndrome, spinal stenosis, or tendon rupture.

The presence of symptoms and previous conditions can be evaluated over telephone, and in their absence, the likelihood of undiagnosed ATTRwt is probably minimal. If the sibling presents red flags for CA, they should be evaluated in accordance with international guidelines. Importantly, as the presented cases demonstrate, the presence of other cardiovascular diseases (e.g. IHD or aortic valve stenosis) should not deter investigation for coexisting CA.

## Data Availability

The data underlying this case report are available and will be provided on request.

## Lead author biography

Dr Oscar Mikael Westin graduated from Umeå University, Sweden, in 2014. He defended his PhD dissertation on CA in 2023 at Copenhagen University, Denmark, and began his cardiology residency shortly thereafter. He has clinical experience in CA from his own screening studies and from working as a subinvestigator for the Apollo-B trial and the Cardio-TTRansform trial.

## Notes

**Consent:** As per COPE and ICMJE recommendations, written consent was obtained from all patients prior to submission of this case report.

**Funding:** None declared.
